# Halotolerant Plant Growth-Promoting Rhizobacteria Isolated From Saline Soil Improve Nitrogen Fixation and Alleviate Salt Stress in Rice Plants

**DOI:** 10.3389/fmicb.2022.905210

**Published:** 2022-06-06

**Authors:** Fiqriah Hanum Khumairah, Mieke Rochimi Setiawati, Betty Natalie Fitriatin, Tualar Simarmata, Saleh Alfaraj, Mohammad Javed Ansari, Hesham A. El Enshasy, R. Z. Sayyed, Solmaz Najafi

**Affiliations:** ^1^Department of Soil Science, University of Padjadjaran, Jatinangor, Indonesia; ^2^Department of Forestry Management, State Agricultural Polytechnic of Samarinda, Samarinda, Indonesia; ^3^Department of Zoology, College of Science, King Saud University, Riyadh, Saudi Arabia; ^4^Department of Botany, Hindu College Moradabad, Mahatma Jyotiba Phule Rohilkhand University Bareilly, Bareilly, India; ^5^Universiti Teknologi Malaysia (UTM), Institute of Bioproduct Development (IBD), Skudai, Malaysia; ^6^Universiti Teknologi Malaysia (UTM), School of Chemical and Energy Engineering, Faculty of Engineering, Skudai, Malaysia; ^7^City of Scientific Research and Technology Applications (SRTA), Alexandria, Egypt; ^8^Department of Microbiology, PSGVP Mandal’s S I Patil Arts, G B Patel Science, and STKVS Commerce College, Shahada, India; ^9^Department of Entomology, Asian PGPR Society for Sustainable Agriculture, Auburn University, Auburn, AL, United States; ^10^Department of Field Crops, Faculty of Agriculture, Van Yüzüncü Yıl University, Van, Turkey

**Keywords:** halotolerant, PGPR, salinity, nitrogen fixation, salt stress, climate change

## Abstract

Salinity is one of the most damaging abiotic stresses due to climate change impacts that affect the growth and yield of crops, especially in lowland rice fields and coastal areas. This research aimed to isolate potential halotolerant plant growth-promoting rhizobacteria from different rhizo-microbiome and use them as effective bioinoculants to improve rice growth under salinity stress conditions. Bioassay using rice seedlings was performed in a randomized block design consisting of 16 treatments (control and 15 bacterial isolates) with three replications. Results revealed that isolates S_3_, S_5_, and S_6_ gave higher shoot height, root length, and plant dry weight compared with control (without isolates). Based on molecular characteristics, isolates S_3_ and S_5_ were identified as *Pseudomonas stutzeri* and *Klebsiella pneumonia.* These isolates were able to promote rice growth under salinity stress conditions as halotolerant plant growth-promoting rhizobacteria. These three potent isolates were found to produce indole-3-acetic acid and nitrogenase.

## Introduction

Soil salinity is a major abiotic stress for plants due to climate change impacts, especially in the agriculture fields around the coastal areas. Global warming causes the sea level to rise due to the melting of glaciers and ice sheets, which encourages saltwater intrusion into the coastal agricultural land (Ullah et al., 2019). Changes in weather patterns like prolonged drought and the increase in average temperature also led to higher evapotranspiration, which positively correlated with increased soil salinity (Bannari and Al-Ali, 2020).

Saline soil has higher amounts of soluble salt (Xiaoqin et al., 2021). Na^+^ is one of the most dominant dissolved salt components because it can form NaCl, Na_2_CO_3_, and Na_2_SO_4_ in the soil (Choudhary and Kharche, 2018). The abundance of Na ions (mostly from NaCl) in saline soil impacts soil’s physical, chemical, and biological properties that prevent plants’ nutrient uptake (Hamid et al., 2021). Soil electrical conductivity value greater than 4 dS/m and the percentage of exchangeable sodium < 15% can be detrimental to plant health, nutrient content, and microbial activity (Diacono and Montemurro, 2015; Numan et al., 2018). On the contrary, essential macronutrients such as nitrogen (N) become hard to be available in soil due to the high concentration of salts (Gondek et al., 2020).

In many cases, plants grown in saline soil often experience diminished root proliferation, failure of seeds germination, reduced photosynthetic activity, and decreased vegetative growth (Gong et al., 2018). Plants experience high osmotic pressure, salt poisoning, and disruption of plant nutrient balance (Shrivastava and Kumar, 2015; Shi et al., 2021). Soil salinity in arid and semiarid areas causes more evapotranspiration than precipitation, creating water stress conditions, and soil minerals undergo a lot of leaching in the plant root zone (Zörb et al., 2019). Limitation of gas exchange, stagnation of stomatal opening and closing, and reduction rate of carbon assimilation due to reduction in plant cellular water potential worsen the plant growth and productivity (Lisar et al., 2012). These disruptions negatively impact global agricultural sustainability (Sunita et al., 2020). This situation warrants sustainable management, cost-effective, and eco-friendly strategies to restore soil fertility in saline ecosystems (Shalaby, 2018; Ansari et al., 2019; Egamberdieva et al., 2019; Niamat et al., 2019; Bhatt et al., 2020; Khumairah et al., 2020; Naveed et al., 2020; Dewi et al., 2021).

These situations demand mitigation strategies and sustenance to alleviate the salinity stress and assist in supplying the nutrients needed for plant growth. Plants cannot be standalone and harbor holobionts inside and outside plant tissues to preserve their growth and development (Vandenkoornhuyse et al., 2015). Plants grown in salinity stress conditions need more support toward these abiotic distresses. Therefore, halotolerant plant growth-promoting rhizobacteria (H-PGPR) isolates as microbiota in plant holobionts support plant growth in saline soil (Setiawati et al., 2020; Sagar et al., 2022a,b). H-PGPR isolates can live, survive, and engage around the root of plants creating a rhizosphere microbiome (Larsen, 1986; Fitriatin et al., 2018). H-PGPR are relatively resistant and tolerant to certain salt levels (Hindersah et al., 2019), i.e., 1–5% NaCl (low halotolerance), 6–18% NaCl (medium halotolerance), and 19–30% NaCl (high halotolerance). H-PGPR balance their cellular osmotic pressure to avoid denaturation caused by salt present in their environment. Thus, they can survive well and benefit the plants more than non-halotolerant (Etesami and Glick, 2020). Inoculating H-PGPR with rice seedlings could significantly increase plant dry weight under salinity stress conditions (Sen and Chandrasekhar, 2014; Abbas et al., 2019; Suriani et al., 2020).

Plant-associated microbial communities are crucial in nutrient availability and plant defense mechanisms to abiotic stress. This research focused on studying the relationship between halotolerant PGPR isolates obtained from saline soil as plant holobionts in the rhizosphere microbiome and evaluating their plant growth-promoting potential to improve rice growth under salinity stress conditions due to climate change impacts.

## Materials and Methods

### Soil Sample Collection

Fifteen rhizosphere soil samples were collected from rice plants, mangroves, and wild grass closest to the shoreline. The soil samples were collected from Sukajaya Village of West Java, Indonesia. This area is Indonesia’s most extensive rice production affected area due to heavy intrusion of seawater. The location map of soil sampling and descriptions of soil sampling location are mentioned in Supplementary Figure 1 and Table 1. Soil samples were separated from plant root residues and dirt. Approximately 300 g of soil sample was put in the sample bag and transported to the laboratory on the same day for isolation and characterization work.

**TABLE 1:** Altitude, coordinate, and elevation of soil sampling locations.

Sample Source	Code	Coordinate		Elevation (m asl)
Rice plant rhizosphere	S_1_	S 6°0′34.157″	E 107°32’02.416″	21
	S_2_	S 6°10′33.349″	E 107°32’01.843″	18
	S_3_	S 6°10′36.759″	E 107°32’01.083″	16
	S_4_	S 6°10′35.694″	E 107°32’01.827″	15
	S_5_	S 6°10′45.948″	E 107°31’56.942″	13
Mangrove rhizosphere	S_6_	S 6°10′28.056″	E 107°31’57.984″	0.15
	S_7_	S 6°10′30.981″	E 107°32′02.333″	16
	S_8_	S 6°10′32.575″	E 107°32′06.392″	16
	S_9_	S 6°10′33.728″	E 107°32′02.684″	19
	S_10_	S 6°10′34.148″	E 107°32′02.305″	20
Wild grass rhizosphere	S_11_	S 6°10′29.348″	E 107°31′55.537″	12
	S_12_	S 6°10′28.231″	E 107°31′57.469″	0.15
	S_13_	S 6°10′30.842″	E 107°32′02.351″	16
	S_14_	S 6°10′34.485″	E 107°32′08.061″	14
	S_15_	S 6°10′34.157″	E 107°32’02.176″	18

### Salinization of Okon Media

Salinized Okon media consisted of maleic acid, K_2_HPO_4_, KH_2_PO_4_, MgSO_4_.7H_2_O, NaCl, agar-agar, and distilled water; the pH was adjusted to 7.0 ± 0.2. Desired EC of salinized Okon media was set using the following equation (Supplementary Figure 2):


y=5.6241+0.0628⁢x

where *y* = desired EC, and *x* is the amount of NaCl added.

In this experiment, 6.0 g of NaCl was added into the Okon media to achieve salinity at 6 dS/m (moderately saline). Salinized Okon media was added into the sterilized Erlenmeyer flask and autoclaved at 1.5 PSI and 121°C for 15 min.

### Salinization of Fahreus Media

For this purpose, Fahreus media containing (g/L) CaCl_2_, MgSO_4_, KH_2_PO_4_, Na_2_HPO_4_.2H_2_O, ferric citrate, yeast extract, microelement, and distilled water was used. For salinization of this media, 6.2 g NaCl was added using a regression equation of *y* = 1.2769 + 0.7666*x* (Supplementary Figure 3.) to maintain a salinity level of 6 dS/m (moderately saline), followed by sterilization at 15 PSI and 121°C for 15 min.

### Isolation of Halotolerant PGPR

Halotolerant PGPR from saline soil were isolated using the plate-dilution frequency technique (Harris and Sommers, 1968). Serial dilutions were made by pipetting 1 ml of soil sample solutions into 9 ml aqua dest (10^–1^) until the dilution series of 10^–5^ was obtained; 10 g of soil samples were added in 90 ml aqua dest followed by stirring and vortexing. At the last dilution, a 0.5 ml suspension was placed into the Petri dish, followed by pouring the salinized Okon media into the Petri dish, then incubated 48–72 h at 27–28°C until the formation of white, convex, and slimy colony appeared. After 72 h of incubation at 27°C, the separated colony was picked up and then subcultured on salinized Okon Media. This activity was repeated three times sequentially to obtain pure isolates, and then, a separated colony from the last streak was preserved (Axler-DiPerte, 2017).

### Screening and Estimation of Plant Growth-Promoting Traits

#### Estimation of Indole Acetic Acid

For the IAA production test, 3 ml of 24 h active culture suspension from each isolate was separately added into each 27 ml liquid Okon media amended with L-tryptophan incubated at 28°C and 100 rpm for 6 days. Salkowski reagent was added in a ratio of 4:1 (supernatant: Salkowski). The mixture was then incubated for 20 min, and absorbance was measured in a spectrophotometer at 535 nm. Following the incubation, 5 ml of liquid culture from each flask was centrifuged at 10,000 rpm for 15 min, and the supernatant was subjected to the estimation of IAA (Chaiharn and Lumyong, 2011).

#### Qualitative and Quantitative Estimation of Phosphate Solubilization

For qualitative estimation of phosphate (P) solubilization, the active culture of each isolate was separately grown on Pikovskaya’s (PKV) agar at 30°C for 48 h and observed for the development of P solubilization zone around the colonies (Pikovskaya, 1948). For quantitative estimation of isolates’ P solubilization, each isolate’s active culture was separately grown in each PKV broth at 30°C, 120 rpm for 48 h, followed by centrifugation at 10,000 rpm for 10 min. The inorganic P in the cell-free supernatant was estimated according to the method of Fiske and Subbarow (1925). Uninoculated PKV agar and PKV broth were used as control.

#### Screening for Production of Ammonia

For screening of ammonia production, each isolate’s active culture was grown in peptone water (PW) medium at 30°C for 24 h. After the incubation, plates were recorded for the occurrence of yellow color (Dutta and Thakur, 2017). Uninoculated PW medium was used as a control.

#### Screening for Production of Siderophore

For screening of siderophore-producing ability, the active culture of each isolate was separately grown on Chrome Azurol S (CAS) agar plates at 30°C for 48 h followed by the development of yellow-orange halos around the colonies (Patel et al., 2018). Uninoculated CAS agar served as a control.

Siderophore production was carried out at shake flask level, and for this, active culture (5 × 10^5^ cells/ml) of each isolate was individually grown in succinate medium (Meyer and Abdallah, 1978) at 30°C for 48 h. This was followed by centrifugation at 10,000 rpm for 10 min, and siderophore content (% siderophore units) from cell-free supernatant was estimated following the CAS shuttle assay (Payne, 1994). An uninoculated SM served as control.

#### Estimation of Nitrogenase Activity

Nitrogenase activity was measured using the acetylene reduction assay (ARA) method (Hellebust and Craigie, 1978). This method involved the incubation process of the material being tested in a gas container containing a partial pressure of acetylene. The pure cultures of halotolerant PGPR isolates were used for quantitative testing by gas chromatography. Before injecting the sample, the gas chromatography device was conditioned for 3 h. Gas chromatography was operated with the initial temperature at 100°C, injector temperature at 150°C, detector temperature at 200°C, and final temperature at 100°C. The type of gas used was nitrogen (40 psi), hydrogen (1.5 kg f/cm^2^), and air (0.5 kg f/cm^2^). The ethylene concentration from each sample was measured by measuring from the area of the ethylene standard. Ethylene standard curves were made in concentrations of 0 μg/ml to 225 μg/ml. The chromatogram results were plotted into an ethylene standard curve; 1 ml of ethylene gas (C_2_H_2_) was injected into each culture tube of halotolerant PGPR and then incubated for 1 h. After incubation, 1 ml of gas from the headspace of each culture tube was taken and subjected to the measurement of the concentration of ethylene (C_2_H_4_) formed using gas chromatography.

### Screening for Salinity Ameliorating Traits

#### Production of Aminocyclopropane-1-Carboxylate Deaminase

For this purpose, active cultures of each isolate were grown in minimal medium (MM) containing (g/L) KH_2_PO_4_, K_2_HPO_4_, MgSO_4_, glucose, and (NH_4_)_2_SO_4_ at 30°C for 48 h followed by observing the growth of the isolate (Safronova et al., 2006). Aminocyclopropane-1-carboxylate deaminase (ACCD) activity from inoculated MM was estimated as per the Penrose and Glick (2003)method. The ACCD activity was defined as the amount of α-keto-butyrate produced per mg of protein per h.

#### Screening for Production of Antioxidant Enzymes

For screening of antioxidant enzymes such as superoxide dismutase (SOD), catalase (CAT), and reduced glutathione oxidase (GSH), each isolate was individually grown in MM at 30°C for 24 h at 120 rpm. This was followed by centrifugation at 1,000 rpm for 10 min to obtain cell homogenate.

In the SOD activity assay, 100 μl of cell homogenate was mixed with 100 μl of pyrogallol solution in EDTA buffer (pH 7.0) followed by measuring the absorbance at 420 nm (Marklund and Marklund, 1974). One unit of SOD was taken as the amount (IU/mg) of SOD required to prevent 50% of the autoxidation of pyrogallol.

In CAT activity, 100 μl of cell homogenate was mixed with 100 μl of hydrogen peroxide (H_2_O_2_) in phosphate buffer (pH 7.0), followed by measuring the absorbance at 240 nm (Beers and Sizer, 1952). One unit of CAT was taken as mM of H_2_O_2_ decomposed/min.

For the GSH assay, 100 μl of cell homogenate was mixed with 100 μl of GSH followed by measuring the absorbance at 240 nm (Nürnberg and Danon, 2016). GSH activity was measured as the reduction in μM of GSH per min.

### Plant Growth Promotion Study in Rice Seedlings—Greenhouse Study

Bioassay test was conducted at the Greenhouse, Faculty of Agriculture, Padjadjaran University, Jatinangor, Indonesia, in March 2020. This experiment aimed at selecting isolates that have the best effect on the growth of rice seedlings. Bioassay tests were conducted using a hydroponic system using salinized liquid Fahreus media. The experiments were conducted in triplicates as a complete randomized block design (RBD), consisting of 16 treatments (control and 15 halotolerant PGPR isolates) with three replications. The rice seed variety used was INPARI-33 sensitive to salinity. Rice seeds were sterilized in HgCl_2_ 0.2% for ± 2 min and in 70% alcohol for ± 2 min, then rinsed with sterile distilled water three times, and then germinated on clean straw paper. Two pieces of straw paper were moistened using salinized distilled water obtained by adding 6 g NaCl to 1 L of distilled water. Seeds were planted, covered with straw paper, and rolled up using plastic. Seed germination was done in an incubator at 28°C for 5 days. After 5 days, rice seedlings’ roots were soaked in salinized liquid Fahreus media and then were transplanted into a 20 mm × 300 mm sterilized test tube, and bacterial suspensions (10^8^ CFU/ml) of liquid salinized Okon media were added. Seedlings’ bodies were supported by sterilized plastic pipes to prevent drowning. Rice seedlings were then stored in test tube racks in the greenhouse. Plant height (cm), root length (cm), and plant dry weight (mg) were recorded at 21 days after planting (DAP). The selection of the best isolates was made using the simple scoring and ranking method.

### Effect of Inoculants on Rice Growth—Pot Experiment

The selected isolates were used as active ingredients for the H-PGPR inoculant in the form of powder using an organic-based carrier (40% peat, 30% compost, 20% biochar, 10% additive). Organic-based carrier was chosen for its characteristics. The nature of organic carriers can have an impact on the effectiveness of rhizobacteria in biofertilizers in supporting plant productivity (Arora et al., 2014). About 35% of bacterial suspension containing 10^9^ CFU/ml was incorporated with the carrier to obtain a bacterial density of about 10^8^ CFU/g.

Simple pot experiment was performed to investigate the effect of H-PGPR inoculant on the abundance of N-fixing bacteria (*Azotobacter* sp. and *Azosprillum* sp.), N uptake, and agronomical traits, and rice yield was done in Cilamaya Wetan, Karawang District (6°15’44”, 107°34’24”, located about 0.5 m above sea level). The soil properties belonged to silty clay texture as an acid soil (pH = 5.04), 2.44% of Org-C, 0.25% of total N, high content of exchangeable Na (2.01 cmol/kg), high salinity (ECe = 6.64 dS/m), and very low base saturation (14.24%).

The experiment was arranged as an RBD consisting of eight treatments, namely, P0 = control; P1 = 500 g SA; P2 = 1000 g SA; P3 = 1500 g SA; P4 = 20 g ST/kg seed; P5 = 20 g ST + 500 g SA; P6 = 20 g ST + 1000 g SA; and P7 = 20 g ST + 1500 g SA. Saline paddy soil from Rawagempol Village, Cilamaya Wetan District, Karawang Regency, from a depth of 0 cm to 25 cm was obtained and then cleaned of plant debris. Then, the soil was placed into a bucket with a capacity of 10 kg. In seed treatment, 20 g of biofertilizers was mixed with rice seeds, followed by soil application according to their respective treatment doses, namely, 0, 500, 1,000, and 1,500 g. In the soil application, biofertilizers were distributed in the soil according to their respective doses without the seed treatment.

The observed responses were the population of N-fixing bacteria, N uptake, and rice’s growth and grain yield. N-fixing bacteria observed were *Azotobacter* sp. and *Azospirillum* sp. *Azotobacter* isolation used the selective Ashby’s nitrogen-free media, and *Azospirillum* isolation used the selective Okon nitrogen-free media. Isolation was carried out by the dilution method. A total of 10 g of soil sample was put into 90 ml of distilled water in a small test tube, then vortexed, made a series of dilutions by pipetting 1 ml of solution into 9 ml of aqua dest and so on until a dilution series of 10^–1^–10^–7^ was obtained; then, 0.1 ml of the dilution was placed into the Petri dish that already contains the Ashby’s and Okon media mentioned earlier and incubated for 48–72 h at room temperature (27–28°C).

Nitrogen uptake in rice plants was analyzed using the Kjeldahl method. An amount of 0.250 g of plant sample was cut into pieces of <0.5 mm in size and placed in a digestion tube; 1 g of selen mixture and 2.5 ml of H_2_SO_4_ p.a. were added into it. The mixture was leveled and left overnight to be stirred. A blank was prepared by adding only 1 g of the selen mixture and 2.5 ml of H_2_SO_4_ p.a. without plant sample into the digestion tube. The next day, it was heated in a digestion block to 350°C. Destruction was complete when white steam comes out and a clear extract was obtained (about 4 h). The tube was removed and cooled, and then, the extract was diluted with ionized water to exactly 50 ml and then vortexed until homogeneous; the tube was left overnight to allow the particles to settle. The clear extract was used for N measurement by distillation or colorimetry.

### Characterization of Potent Isolates

#### Phenotypic Characterization

Selected halotolerant PGPR isolates were characterized based on their morphological traits and biochemical activity. Morphological characterization consisted of colony characteristics and Gram staining (Hucker and Conn, 1923). At the same time, biochemical characterization involved the measurement of IAA production and nitrogenase enzyme activity.

#### Molecular Characterization

Molecular identification of potent H-PGPR isolates was carried out based on phylogenetic analysis of 16s rRNA gene sequencing. The isolates were grown in Luria Bertani Broth overnight at 30°C at 120 rpm followed by centrifugation at 10,000 rpm for 2 min to obtain the cell pellets. The 16S rRNA gene of the isolates was amplified with universal primers 16S-27F (5′AGAGTTTGATCCTGGCTCAG3′) and 16S-1492R (5′GGTTACCTTGTTACGACTT3′) followed by polymerase chain reaction (PCR) and gel electrophoresis on a 0.8% agarose gel. The 16S rRNA gene sequence was analyzed using the 16S rRNA gene amplicon sequencing on ABI 3730Xl automated sequencer using a ready reaction kit (Perkin Elmer Applied Biosystems Division, CA, United States). Phylogenetic trees were constructed with the help of the neighbor-joining method using MEGA5 software. H-PGPR isolates were identified based on their phylogenetic relationship with the standard database of NCBI (Cole et al., 2009).

### Statistical Analysis

All the experiments were performed in triplicates, and a mean of triplicate was further analyzed statistically using the Statistical Analysis System (SAS Institute, North Carolina State University). *F*-test was performed to show significant effects on tested variables. Finally, Duncan multiple range test (DMRT) was performed (*p* < 0.05) (Gomez and Gomez, 1984).

## Results

### Isolated Potent Halotolerant PGPR

Fifteen H-PGPR isolates were obtained from rice, mangroves, and wild grass rhizosphere from the composite soil samples. These isolates were then screened for IAA production and nitrogenase activity.

### Plant Growth-Promoting Traits of Potent H-PGPR

#### Indole Acetic Acid

All H-PGPR isolates can produce varying amounts of IAA. Isolate S_5_ had the highest amount of IAA compared with S_3_ and S_6_. It produced 0.648 μg/ml IAA *vis-à-vis* 0.592 μg/ml produced by isolates S_3_ and S_6_ (Table 2).

**TABLE 2:** Screening and the production of various plant growth promoting and salinity ameliorating of Halotolerant PGPR isolates.

Traits	Isolates		
	S_3_	S_5_	S_6_
P solubilization index	10.1 ± 0.41	7.1 ± 2.21	6.2 ± 3.35
P solubilization (μg/mL)	4102 ± 7.41	3021 ± 2.01	2865 ± 3.51
Ammonia production	+++	++	++
Siderophore production	81.2 ± 0.02	73.5 ± 0.03	69.8 ± 0.02
ACCD activity (μM/mg^/^h)	0.952 ± 0.02	0.818 ± 0.01	0.798 ± 0.03
SOD activity (IU/mg protein)	14.79 ± 0.03	13.01 ± 0.01	10.96 ± 0.02
CAT activity (IU/mg protein)	0.095 ± 0.02	0.087 ± 0.01	0.079 ± 0.03
GSH activity (μg/mg protein)	27.21 ± 0.01	23.82 ± 0.02	21.36 ± 0.03

*+ = present − = absent, ++ = positive, +++ = strong positive, % SU = % siderophore units. Values are the average of triplicates and were analyzed by Duncan Multiple Range Test at 5% real level.*

#### Phosphate Solubilization

Isolate S_3_ showed a maximum P solubilization zone on the PKV agar plate compared with the isolates S_5_ and S_6_. The isolate S_3_ exhibited a maximum P solubilization index (10.1 mm) compared with 7.1 mm and 6.2 mm P solubilization index by S_5_ and S_6_, respectively (Table 2).

#### Production of Ammonia and Siderophore

All three isolates produced varying amounts of ammonia and siderophore. However, the isolate S_3_ yielded maximum ammonia (+++) and siderophore units (81.2% SU) compared with S_5_ and S_6_ (Table 2).

#### Nitrogenase Activity

In each isolate, nitrogenase activity was directly proportional to nitrogenase concentration. Isolate S_3_ had the highest nitrogenase activity (3.207 μM/ml/h), while isolates S_5_ and S_6_ showed 2.217 μM/ml/h nitrogenase activity, respectively. Thus, the S_3_ isolate had the highest nitrogenase enzyme productivity as it had the highest nitrogenase concentration. This isolate also showed more plant growth-promoting effects in rice seedlings compared with other isolates and control (Table 2).

### Salinity Ameliorating Traits

#### Aminocyclopropane-1-Carboxylate Deaminase

All three potent isolates produced varying amounts of ACCD. However, isolate S_3_ exhibited more ACCD activity than isolates S_5_ and S_6_ (Table 2).

#### Antioxidant Enzymes

All three potent isolates produced varying amounts of antioxidant enzymes such as SOD, CAT, and GSH. However, isolate S_3_ exhibited maximum activities of these enzymes compared with isolates S_5_ and S_6_ (Table 2).

### Plant Growth Promotion in Rice Seedlings Under Salinity Stress

The effects of inoculation of H-PGPR isolates on plant growth were evident on plant height, root length, and plant dry weight of rice seedlings at 21 DAP. All H-PGPR isolates could promote plant height even though not significantly different from control (without isolates) (Table 3). The inoculation of H-PGPR isolates in rice seedlings showed a significant (*p* < 0.05) improvement in the growth of rice plants under salinized Fahreus media (6 dS/m, moderately saline). Isolate S_3_, S_5_, and S_6_ resulted in 318% (9.13 cm) improvement in plant height, 56.69% (12.17 cm) increase in root length, and 73.18% (27.33 mg) improvement in plant dry weight. The inoculation of H-PGPR in rice-to-rice plants could promote plant growth under saline conditions. Isolates were also proven to have halotolerant abilities (tolerant to salinity), where they were able to survive and even increase plant growth in saline conditions.

**TABLE 3:** Effect of halotolerant PGPR isolates on growth parameters in rice seedlings at 21 Days After Planting.

Treatments	Code	IAA (μg/mL)	Nitrogenase activity (μM/mL/h)	Plant height (cm)	Root length (cm)	Plant dry weight (mg)
Control (without isolates)	S_0_	0.000	0.000	2.87 ± 0.67^ab^	6.90 ± 0.46^a^	20.00 ± 2.00^abc^
H-PGPR isolate from rice plant 1	S_1_	0.387^ab^	2.018^ab^	2.73 ± 1.01^ab^	9.77 ± 2.86^abc^	15.67 ± 2.08^a^
H-PGPR isolate from rice plant 2	S_2_	0.317^ab^	2.200^ab^	4.50 ± 4.52^ab^	10.30 ± 2.26^abc^	16.67 ± 3.51^ab^
H-PGPR isolate from rice plant 3	S_3_	0.592^ab^	3.207^abc^	9.13 ± 7.07^b^	12.07 ± 1.27^d^	33.34 ± 8.14^d^
H-PGPR isolate from rice plant 4	S_4_	0.328^ab^	2.001^ab^	4.27 ± 2.25^ab^	10.47 ± 1.19^abc^	21.33 ± 3.06^abc^
H-PGPR isolate from rice plant 5	S_5_	0.648^abc^	2.217^ab^	6.13 ± 2.61^ab^	12.17 ± 0.70^d^	27.33 ± 3.06^cd^
H-PGPR isolate from mangrove 1	S_6_	0.592^abc^	2.217^ab^	6.50 ± 5.20^ab^	11.17 ± 1.07^bc^	24.67 ± 8.14^bc^
H-PGPR isolate from mangrove 2	S_7_	0.293^ab^	1.117^a^	5.63 ± 2.56^ab^	8.57 ± 1.05^abc^	20.34 ± 2.31^abc^
H-PGPR isolate from mangrove 3	S_8_	0.292^ab^	1.182^ab^	5.10 ± 1.47^ab^	8.73 ± 1.95^abc^	18.67 ± 2.08^ab^
H-PGPR isolate from mangrove 4	S_9_	0.308^abc^	1.322^abc^	4.77 ± 2.83^ab^	10.93 ± 1.85^bc^	21.66 ± 1.53^abc^
H-PGPR isolate from mangrove 5	S_10_	0.273^ab^	1.121^ab^	2.33 ± 0.21^a^	10.00 ± 0.70^abc^	19.67 ± 3.21^abc^
H-PGPR isolate from wild grass 1	S_11_	0.281^ab^	1.101^a^	4.13 ± 3.48^ab^	10.63 ± 1.91^bc^	22.00 ± 2.00^abc^
H-PGPR isolate from wild grass 2	S_12_	0.301^ab^	1.481^ab^	5.30 ± 2.85^ab^	9.17 ± 1.10^abc^	19.33 ± 1.53^abc^
H-PGPR isolate from wild grass 3	S_13_	0.199^b^	1.332^abc^	4.60 ± 1.39^ab^	10.80 ± 2.69^bc^	22.66 ± 0.58^abc^
H-PGPR isolate from wild grass 4	S_14_	0.232^a^	1.411^ab^	5.50 ± 3.39^ab^	9.00 ± 2.46^abc^	23.34 ± 3.06^abc^
H-PGPR isolate from wild grass 5	S_15_	0.187^b^	1.033^ab^	5.60 ± 2.86^ab^	8.13 ± 3.23^ab^	22.67 ± 2.52^abc^

*Figures are the mane of triplicates. Figures followed by the same notation are not significantly different based on Duncan Multiple Range Test at 5% real level. Figures followed by the same letter are not significantly different.*

**FIGURE 1:**
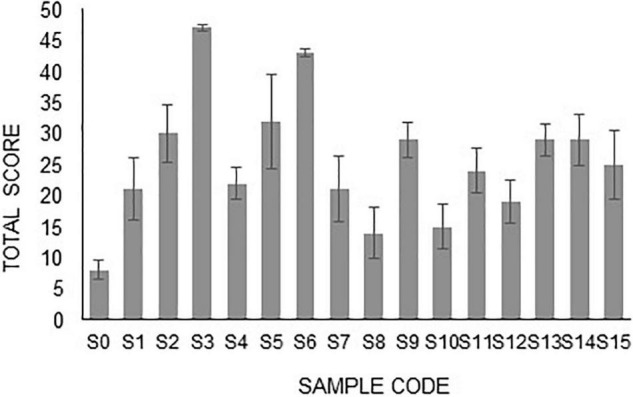
Scoring of H-PGPR isolates based on plant height, root length, and plant dry weight.

The best H-PGPR isolates were selected using simple scoring and ranking methods based on plant height, root length, and dry weight (Herdiyantoro et al., 2018). The rules in the simple scoring and ranking method were as follows: (i) the lowest plant height was given a score of 1, the higher was given a score of 2 and so forth; (ii) the lowest root length was given a score of 1, the higher was given a score of 2 and so forth; (iii) the lowest plant dry weight was given a score of 1, the higher was given a score of 2 and so forth; (iv) all scores were summed up, and ranking was done based on the highest of the total score; (v) the highest of the total score was rank 1, the lower of the total score ranked 2 and so forth. Isolates S_3_, S_5_, and S_6_ had the highest score, sequentially ranking 1, 2, and 3. These three isolates were used for morphological traits and biochemical activity characterization (Figure 1).

### Effect of H-PGPR Inoculant on the Abundance of N Fixer in Rice Rhizomicrobiome

The abundance of *Azotobacter* sp. and *Azospirillum* sp. was increased significantly by the seed treatment (20 g inoculant/kg) and increased dosage of inoculant (Table 4). The highest population of *Azotobacter* sp. (2.80 × 10^7^ CFU/g soils) and *Azospirillum* sp. (2.13 × 10^7^ CFU/g soils) were obtained by the seed treatment with 20 g inoculant/kg combined with 1,500 g inoculant/ha of soil application. The increment was 110.5% and 238.1%, respectively, higher than the control. The *Azotobacter* sp. or *Azospirillum* sp. of inoculated pots with 20 g/kg seed and 1,500 g/ha (P_5_) was still significantly higher than treated pots with 1,500 g/ha of inoculant (P_3_). Even though *Azospirillum* sp. population was not significantly different with treated pots with 1,500 g/ha of inoculant (P_3_), the increment compared with the control was lower (only 174.6%) than P_5_ (238.1%). These results indicated that the introduced inoculant could adapt and multiply in rhizomicrobiome.

**TABLE 4:** Effect HNF PGPR inoculant as seed treatment (ST = 20 g/kg seed) combined soil application (SA = g/ha) on the abundance of N fixers and PGPR (*Azotobacter* sp and *Azospirillum* sp) in rice rhizomicrobiome.

Inoculant Application	*Azotobacter sp* (x 10^7^ CFU)	Increment (%)	*Azospirillum* sp (x 10^8^ CFU)	Increments (%)
P_o_ = control	1.33 ab	–	0.63 a	–
P_1_ = 500 g SA	1.37 ab	3.0	0.80 ab	27.0
P_2_ = 1000 g SA	1.55 b	16.5	1.23 bc	95.2
P_3_ = 1500 g SA	1.73 c	30.5	1.73 dc	174.6
P_4_ = 20 g ST/kg seed. mg/plant	1.30 a	−2.3	0.70 a	11.1
P_5_ = 20 g ST + 500 g SA	1.53 b	15.0	1.30 bc	106.3
P_6_ = 20 ST + 1000 g SA	1.77 c	33.1	1.63 cd	158.7
P_7_ = 20 ST + 1500 g SA	2.80 d	110.5	2.13 d	238.1

*Average value followed by the same letter within same column were not different significantly DMRT 0.05%.*

### Effect of H-PGPR Inoculant on N Uptake, Growth Characters, and Rice Yield

The N uptake and agronomical traits (plant height and a number of tillers at 50 DAP (Table 5), and yield component and harvested rice grain (Table 6), were significantly influenced by the seed treatment (ST) with 20 g/kg of seed combined with 500–1,500 g/ha of H-PGPR inoculant. The N content and status of plant tissue were improved significantly by applying inoculant. Despite N, the status belongs to the optimal condition, but the measured value tends to be increased as shown by the visual crop performance (the leaf of the treated plot is greener than control). The enlarged dosage of H-PGPR inoculant increased the N uptake, plant height, and the number of tillers significantly. In contrast, applying H-PGPR inoculant as seed treatment increased the number of tillers, while the N uptake and plant height were affected considerably. Briefly, the combined effects of seed treatment and soil application on the measured responses were higher than the control, but not different from the obtained result with soil application of inoculant. These results indicated that the soil application of 1,000–1,500 g/ha of H-PGPR inoculant significantly increased the N uptake, plant height, number of tillers, and rice grain yield. The highest rice grain yield was obtained by applying 1,500 g/ha of H-PGPR inoculant (35.1 g/plant or 6.4 ton/ha) or in combination with 20 g/kg seed treatment (38.9 g plant or 7.0 ton/ha). Compared with the control, rice grain yield was increased by 41.1–161.1% by the soil application of 500–1,500 g/ha of inoculant. Moreover, applying 20 g/kg seed of inoculant combined with 500–1,500 g/ha increased the rice grain yield by 57.4–189.4% but not significantly different with soil application only.

**TABLE 5:** Effect of HNR PGPR inoculant as seed treatment (ST) and soil application (SA) on growth component (the N-uptake, plant height and number tiller of rice at 50 DAP) on saline soils.

Inoculant Application (ST = 20 g/kg seed. SA = g/ha)	N-Uptake		Plant height (50 DAP)	Tiller (tiller/clump)
	(%)	mg plant^–1^		
P_o_ = control	2.24 a	2.77 a	79.40 a	20.00 a
P_1_ = 500 g SA	2.78 a	3.31 abc	81.91 bc	24.42 bc
P_2_ = 1000 g SA	2.84 a	4.33 cd	83.74 cd	26.83 cd
P_3_ = 1500 g SA	2.91 a	5.05 d	86.22 e	34.50 e
P_4_ = P4 = 20 g ST/kg seed. mg/plant	2.50 a	2.98 ab	79.79 ab	23.08 b
P_5_ = 20 g ST + 500 g SA	2.73 a	3.74 abcd	82.85 cd	27.08 cd
P_6_ = 20 ST + 1000 g SA	2.78 a	4.23 bcd	84.98 de	29.67 d
P_7_ = 20 ST + 1500 g SA	2.83 a	4.65 d	86.97e	32.58 e

*Average value followed by the same letter within same column were not different significantly DMRT 0.05%.*

**TABLE 6:** Effect of HNR PGPR inoculant on rice component and grain yield on saline soils.

Inoculant Application	panicles/clump	nr.grain/panicle	Weight of 1000 grain (g)	Grain yield		
				g/clump	Growth (%)	t/ha
P_o_ = control	13.2 a	79.0 a	24.0 a	13.5 a		2.4
P_1_ = 500 g SA	14.6 ab	92.6 b	24.7 ab	19.1 b	41.8	3.4
P_2_ = 1000 g SA	16.2 bc	99.4 bc	25.6 ab	25.5 c	90.0	4.6
P_3_ = 1500 g SA	18.6 d	109.8 cd	25.9 bc	35.1 d	161.0	6.3
P_4_ = 20 g ST/kg seed	14.4 ab	77.9 a	24.4 ab	15.4 a	14.3	2.8
P_5_ = 20 g ST + 500 g SA	15.4 bc	91.9 b	25.5 ab	21.2 b	57.4	3.8
P_6_ = 20 ST + 1000 g SA	17.0 cd	102.3 bcd	26.0 bc	29.2 c	116.9	5.3
P_7_ = 20 ST + 1500 g SA	18.6 d	113.7 d	27.6 c	38.9 d	189.4	7.0

*Average value followed by the same letter within same column were not different significantly DMRT 0.05%.*

A comparative result for soil application and seed treatment was done to determine the best technique application between treatments (Table 7). The population of N fixers (*Azotobacter* sp. and *Azospirillum* sp.) was slightly higher at the seed treatment application, but N uptake, plant height, number of tiller, panicles/clump, number of grain/panicle, weight of 1,000 grain, and grain yield showed a better performance on soil application. Results showed that H-PGPR biofertilizer was better to be applied in soil than as seed treatment.

**TABLE 7:** Biochemical activities of H-PGPR isolates.

Isolate Code	Characteristics					
	Shape	Elevation	Color	Cell	Gram staining character	Growth in Okon media (6 dS/m)
S_3_	rounded	*convex*	white	*coccus*	Gram Negative	0.592 ± 0.027
S_5_	rounded	*convex*	white	*coccus*	Gram Negative	0.648 ± 0.107
S_6_	Rounded	*convex*	white	*coccus*	Gram Negative	0.592 ± 0.027

*Figures are the mane of triplicates. Analyzed for standard deviation (SD).*

### Characteristics of Selected Potent H-PGPR Isolates

The morphological traits and biochemical characteristics showed that all three potent H-PGPR isolates (S_3_, S_5_, and S_6_) are Gram-negative rods (Table 7) that can survive under moderate salinity conditions.

**FIGURE 2:**
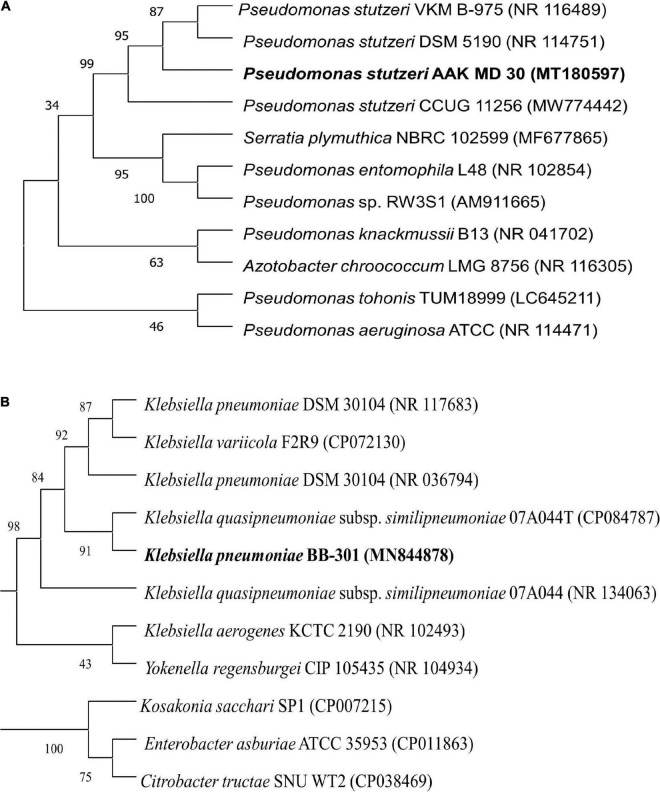
Phylogenetic analysis of H-PGPR isolates, **(A)** *Pseudomonas stutzeri* and **(B)** *Klebsiella pneumonia*, based on 16s rRNA gene sequence homology drawn using the neighbor-joining method (MEGA 5.0 software) with evolutionary distances computed using Kimura’s two-parameter model.

Among these three isolates (S_3_, S_5_, and S_6_), two isolates (S_3_ and S_5_) were subjected to molecular identification as these isolates appeared as potent multifarious PGPR. Isolate S_3_ showed 98.06% similarity with *Pseudomonas stutzeri* (Figure 2A), while isolate S_5_ resembled 100% with *Klebsiella pneumonia* (Figure 2B); 16s rRNA gene sequences of these isolates were submitted to the NCBI gene bank under the accession numbers SUB11206984 and SUB11207011, respectively.

## Discussion

Saline soils are known to harbor halophilic rhizobacteria. This study reports that *P. stutzeri* and *K. pneumoniae* isolated from rice plant rhizosphere were H-PGPR that can improve the growth of rice seedlings under salinity stress conditions due to climate change impacts.

Halotolerant rhizobacteria exert many beneficial effects on plant growth and help in ameliorating soil salinity (Saxena et al., 2013; Sagar et al., 2020a,b; Kapadia et al., 2021; Kusale et al., 2021a).

*Pseudomonas stutzeri* and *K. pneumoniae* used in this study were able to produce IAA, nitrogenase enzyme, P solubilization, ammonia, and siderophore as a force to help plant growth and mitigate salinity stress in plants. These findings were in line with the fact that PGPR provide a range of benefits to the plants (Baba et al., 2021), such as plant growth promotion through the production of phytohormones (Kalam et al., 2020), nitrogen fixation (Kusale et al., 2021a), P solubilization (Sharma et al., 2013, 2016), ammonia production (Kusale et al., 2021a), and siderophore production (Patel et al., 2016; Shaikh et al., 2016; Khan et al., 2019; Sayyed et al., 2019; Jabborova et al., 2020; Basu et al., 2021). They also produce various metabolites that protect the plant from oxidative damages exerted by salinity stress (Sagar et al., 2019, 2020a,2020b,2022a,2022b; Jabborova et al., 2020).

Salt tolerance in *P. stutzeri* and *K. pneumonia* is considered a strategy for organisms’ survival and growth under saline conditions. In this study, *P. stutzeri* and *K. pneumoniae* produced ACCD and antioxidant enzymes. This was a novel finding that two potent isolates were identified as agents in mitigating salinity stress for their salinity ameliorating traits abilities.

Salt-tolerant bacteria limit high amounts of salt into the cell through cell membranes or walls. The cell membranes or cell walls of halophilic bacteria have a specific composition that is accurately resistant to high salt concentrations. Osmotic adaptation in these bacteria helps them regulate the intracellular ionic concentration by pumping out the Na^+^/K^+^ ions using antiporter or K^+^/Na^+^ ion transporters. After that, bacteria accumulate the compatible solutes by endogenous biosynthesis and upregulation of the synthesis of essential amino acids, proteins, and enzymes (Noori et al., 2018). These bacteria are well known as N fixer and PGPR, which contribute to nutrients availability, plant health, plant growth, and salinity stress (Yao et al., 2010; Simarmata et al., 2018; Shultana et al., 2020).

Several scientists had examined and supported the findings of this study that the rhizobia are more tolerant to salinity stress compared with their host plant, but the growth and survival vary under saline conditions depending on the strains and their salt tolerance threshold. Kusale et al. (2021a) isolated multifarious halotolerant *Klebsiella variicola* SURYA from the wheat rhizosphere. The isolate could grow in the presence of a high salt concentration (160 mM). Production of IAA was later found to be one of the principal salinity ameliorating components in this isolate. Noori et al. (2018) isolated *Klebsiella* sp., *Kosakonia cowanii*, and *Sinorhizobium meliloti* and identified these isolates as salt-tolerant bacteria. These isolates could tolerate up to 1,200 mM NaCl, fix nitrogen, solubilize phosphorous, produce IAA, siderophore, HCN, and ACC deaminase enzyme. Sapre et al. (2018) reported that *Klebsiella* sp. enhanced 20% plant biomass under saline stress conditions with respect to negative control seedlings. Kusale et al. (2021a) also affirmed that inoculation of halotolerant *K. variicola* improved plant growth parameters, i.e., roots, shoots, and chlorophyll content. *P. stutzeri* was also proven to increase tomatoes’ plant fresh and dry weight under salinity stress (Samosir et al., 2019).

The H-PGPR is classified as diazotrophic bacteria such as *Rhizobium, Azotobacter*, and *Azospirillum* that can produce IAA with or without tryptophan precursors (Fazeli-Nasab and Sayyed, 2019). H-PGPR inoculation can provide nutrients for plants and increase plant growth in the vegetative phase. This statement indirectly indicates that plant growth was influenced by the ability of each isolate to fix nitrogen and make it available for the plant to uptake as plant holobionts. Several halotolerant rhizobacteria, including *Pseudomonas* sp. and *Klebsiella* sp., produce various plant beneficial metabolites such as phytohormones (Kapadia et al., 2021) and antioxidant enzymes (Arora et al., 2020). *Halotolerant* sp. also increases the value of ARA and the production of the IAA, which was analyzed under a salt concentration of 0.3 M NaCl (± 30 d S/m) (Paul et al., 2014).

The H-PGPR can balance osmotic pressure to avoid denaturation caused by salt in the environment by accumulating salt and osmolytes (organic molecules) in their system (Albaladejo et al., 2017). Inoculating PGPR isolates to crops helps convert the insoluble nutrients into soluble nutrients, making them available to the plants (Etesami and Glick, 2020). N-fixing halotolerant rhizobacteria can maintain their growth-promoting attributes even under saline conditions (Ding et al., 2005). *P. stutzeri* and *K. pneumonia* have long been known for their N-fixing ability. Rice seedlings’ growth with the inoculation of *P. stutzeri* A15 resulted in better performance compared with chemical nitrogen fertilization (Reetha et al., 2014). *K. variicola*, which is identified as a N-fixing species, also acts as N-fixing rhizobacteria (Chen et al., 2016; Pham et al., 2017; Kumar et al., 2020).

Rhizobacteria can act as stimulants and produce hormones such as auxins and gibberellins to help promote plant growth (Jain et al., 2021). Salinity stress can inhibit enzyme activity and cause metabolic changes in plant cells due to the accumulation of too high salts in the cytoplasm. The concentration of cytokinin and auxin hormones decreases, while the concentration of ethylene and abscisic acid increases (Wani et al., 2016). However, the three isolates in this experiment (S_3_, S_5_, and S_6_) were able to produce IAA hormone and nitrogenase enzyme, which increased the vegetative plant growth.

Biochemical attributes of rhizobacteria to produce certain hormones, organic acid, and/or enzymes are beneficial for plant growth under salinity stress (Duarte et al., 2020). Production of IAA is directly proportional to the levels of tryptophan given. Tryptophan functions as a precursor to IAA, yet bacteria also can produce IAA (Cavalcante da Silva et al., 2020). Under salinity stress conditions, plants will increase the ABA content and decrease the IAA content (Thairu et al., 2014). In addition, salinity stress can also disrupt bacterial metabolism and is toxic to plants (Saxena et al., 2013). The nitrogenase activity test performed using the ARA method has high accuracy because it can detect up to a concentration of 0.001 μM (Isayenkov and Maathuis, 2019). Rhizobacteria fix nitrogen to meet their needs in the formation of nucleic acids, and when nitrogen needs are met, excess nitrogen is released into the rhizosphere for use by plant roots (Bhutani et al., 2018).

Salinity impairs nutrient balance and causes nutrient deficiency due to the competition between Na^+^ and Cl^–^ with soil nutrients such as K^+^, Ca^2+^, and NO_3_^–^ (Hmaeid et al., 2019; Kapadia et al., 2021). Salt ions such as Na^+^ and Cl^–^ also cause chloroplasts of plants to experience lysis due to high salt concentrations and degradation of leaf tissue cells (Saxena et al., 2013). Abundance and microbial biodiversity of rhizomicrobiome due to the application of H-PGPR inoculant (*P. stutzeri* or *K. pneumonia*) and increasing population of other beneficial PGPR play an important role in increasing the availability of growth factors and nutrients for supporting the rice growth and development (Yao et al., 2010; Benaissa et al., 2019; Lami et al., 2020). The presence and domination of beneficial microbes in rhizomicrobiome improve the soil and plant health, enhance rice growth, and enhance rice productivity on saline soils (Cavite et al., 2021; Daulay and Simarmata, 2021; Simarmata et al., 2021). Plant growth can be affected by the availability of nutrients, environmental conditions, and physiological processes that occur in plants (Kusale et al., 2021a). In addition, the application of PGPR combined with the application of ameliorant (compost, dolomite) or organic fertilizers could improve the effectiveness of microbial fertilizers or biofertilizers (Simarmata et al., 2016; Simarmata et al., 2019; Shilev, 2020).

The PGPR are known to ameliorate salt stress through the production of ACCD (Sagar et al., 2020a,b). Halophiles adapted to salt stress excrete a wide range of PGP metabolites (Hamid et al., 2021; Khan et al., 2021) and various stress-tolerant enzymes (Kusale et al., 2021b). Sagar et al. (2020a) reported the production of various PGP traits and ACCD in *E.cloacae* PR4. Jabborova et al. (2020) found that halophilic endophytes produce various PGPR traits.

Production of ACCD by PGPR is the major mechanism of salinity stress tolerance (Shrivastava and Kumar, 2013; Sagar et al., 2020a). The enzyme ACCD lowers the level of ACC in root exudates; the suboptimal level of ACC reduces the concentration of ethylene in the plant roots and thus helps in root length, which improves the absorption of nutrients (Kusale et al., 2021a; Sagar et al., 2022b). A wide range of ACCD-producing PGPR, including *Klebsiella* sp. and *Pseudomonas* sp., ameliorate various stresses, including salinity stress in plants (Acuña et al., 2019). *Klebsiella* sp. has been reported to produce ACCD (Kusale et al., 2021a). These isolates grew well at high salt levels, showed optimum ACCD activity at high salt levels, and helped ameliorate salt stress in crops.

Salinity conditions create oxidative stress that damages the cell membranes and cell structures in microbes and plant cells. PGPR produce various antioxidant enzymes such as SOD, CAT, and GSH (Acuña et al., 2019). These enzymes protect plants from oxidation due to osmotic shocks caused by salt stress (Fazeli-Nasab and Sayyed, 2019). Under salt stress conditions, the presence of antioxidant enzyme-producing rhizobacteria activates an antioxidative defensive system in the crops and helps remove the free radicals produced due to salt (Acuña et al., 2019). Sapre et al. (2018) reported halophilic *Klebsiella* sp. that tolerated high salt concentration and produced antioxidant enzymes under salt stress conditions.

Plant height and root length depend on nitrogen availability and are also influenced by the ability of each isolate to produce plant growth-promoting metabolites to improve plant growth (Khumairah et al., 2018). The ability of rhizobacteria to increase plant growth depends on the type of rhizobacteria and their respective abilities. Rhizobacteria that produce multiple metabolites and in higher concentrations provide more nutrients to the plants and thus help grow plants. According to Gupta et al. (2019), each isolate has different abilities in increasing plant growth. The ability of rhizobacteria to increase plant growth and yield depends on the type of rhizobacteria itself (Egamberdieva et al., 2019; Khairnar et al., 2022). *Zhihengliuella halotolerant* strain A1B62 and *Brachybacterium* sp. strain B0sh64 showed longer fresh root and heavier shoot fresh weight of *Suaeda maritima* compared with other strains and control (Alishahi et al., 2020).

Most of the saline soils along coastal areas have a low organic matter content and low fertility. Consequently, an integrated crop and soils management by planting adapted and saline-tolerant rice variety combined with ameliorant application and managing the biodiversity of microbe (microbial fertilizers) as environmentally friend fertilizers are required for rhizomicrobiome engineering to improve soil health, nutrient status and availability, fertilizers efficiency, crop growth and productivity, and alleviate salinity stress.

## Conclusion

The salinity of agriculture is the major damaging stress that negatively impacts the growth and yield of crops, including rice. The physicochemical approaches to combat soil salinity have fewer successes and more harmful effects. The use of rhizobacteria that can tolerate high salt concentration while producing beneficial plant metabolites can serve as effective bioinoculants to improve rice growth under salinity stress conditions and help in salinity amelioration. This study reveals that halotolerant *P. stutzeri* and *K. pneumonia* produce a wide range of PGP metabolites and antioxidant enzymes that help crop plants to grow under salinity stress. These isolates can be used as potent bioinoculants for improving rice growth in saline soil. Due to climate change impacts, it can be further developed as a new biogenic agent to alleviate salinity stress in rice cultivation.

## Data Availability Statement

All data presented in the study are included in the article/Supplementary Material, further inquiries can be directed to the corresponding author/s.

## Author Contributions

FK and TS: conceptualization. FK, MS, RS, and BF: methodology and formal analysis. HE, SA, and TS: fund acquisition. FK and RS: writing the original draft. FK, MA, RS, TS, HE, and SN: writing-review and editing. All authors have read and agreed to the published version of the manuscript.

## Conflict of Interest

The authors declare that the research was conducted in the absence of any commercial or financial relationships that could be construed as a potential conflict of interest.

## Publisher’s Note

All claims expressed in this article are solely those of the authors and do not necessarily represent those of their affiliated organizations, or those of the publisher, the editors and the reviewers. Any product that may be evaluated in this article, or claim that may be made by its manufacturer, is not guaranteed or endorsed by the publisher.
