# Erratum: Halotolerant plant growth-promoting rhizobacteria isolated from saline soil improve nitrogen fixation and alleviate salt stress in rice plants

**DOI:** 10.3389/fmicb.2022.1107282

**Published:** 2022-12-06

**Authors:** Frontiers Production Office

**Affiliations:** Frontiers Media SA, Lausanne, Switzerland

**Keywords:** halotolerant, PGPR, salinity, nitrogen fixation, salt stress, climate change

Due to a production error, the author **Mohammad Javed Ansari** was linked to affiliations three and four. This should be corrected to affiliation four only.

The publisher apologizes for this mistake. The original version of this article has been updated.

