# Supplementary figures and images for: Halotolerant Plant Growth-Promoting Rhizobacteria Isolated From Saline Soil Improve Nitrogen Fixation and Alleviate Salt Stress in Rice Plants
Source: Front Microbiol. 2022 Jun 6;13:905210. doi: 10.3389/fmicb.2022.905210 (PMC9236307; doi:10.3389/fmicb.2022.905210)

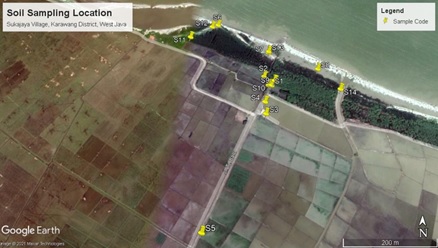

Supplement: Supplementary file 1 [file Image_1.JPEG]

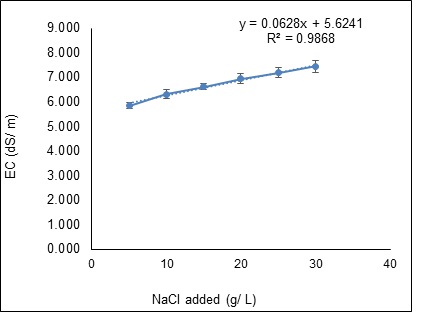

Supplement: Supplementary file 2 [file Image_2.JPEG]

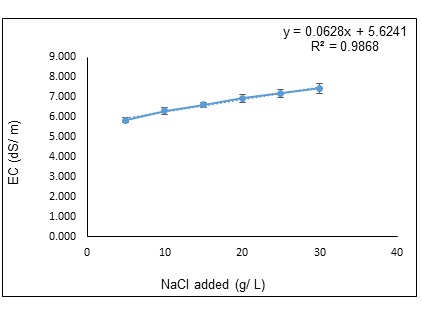

Supplement: Supplementary file 3 [file Image_3.JPEG]
